# Paternal predictors of the mental health of children of Vietnamese refugees

**DOI:** 10.1186/1753-2000-5-2

**Published:** 2011-01-10

**Authors:** Aina B Vaage, Per H Thomsen, Cécile Rousseau, Tore Wentzel-Larsen, Thong V Ta, Edvard Hauff

**Affiliations:** 1 Centre for Child and Adolescent Mental Health, Uni Health, University of Bergen, Norway; 2 Department of Child and Adolescent Psychiatry, Stavanger University Hospital, Box 8100, 4068 Stavanger, Norway; 3 Centre for Child and Adolescent Psychiatry, University of Aarhus, Bup Hospital, Harald Selmersvej 66, 8240 Risskov, Denmark; 4 Division of Social and Cultural Psychiatry, McGill University, CLSC Parc Extension, 7085 Hutchison, Montreal QC, H3N 1Y9, Canada; 5 Centre for Clinical Research, Haukeland University Hospital, Armauer Hansens hus, 5021 Bergen, Norway; 6 International House Foundation, Sandvikveien 13, 4016 Stavanger, Norway; 7 Institute of Clinical Medicine, Faculty of Medicine, University of Oslo, Box 1130 Blindern, 0318 Oslo, Norway; 8 Division of Mental Health and Addiction, Oslo University Hospital, Norway

## Abstract

**Background:**

Intergenerational transmission of trauma as a determinant of mental health has been studied in the offspring of Holocaust survivors and combat veterans, and in refugee families. Mainly negative effects on the children are reported, while a few studies also describe resilience and a possible positive transformation process. A longitudinal prospective cohort study of Vietnamese refugees arriving in Norway in 1982 reports a 23 years follow-up, including spouses and children born in Norway, to study the long-term effects of trauma, flight, and exile on the offspring of the refugees.

Objectives of the study:

1. To study the association between the psychological distress of Vietnamese refugee parents and their children after 23 years resettlement.

2. To analyse paternal predictors for their children's mental health.

**Methods:**

Information from one or both parents at arrival in 1982 (T1), at follow-up in 1985 (T2), and 23 years after arrival (T3) was included. The mental health was assessed by the Global Severity Index (GSI) of the self-report Symptom Check List-90-R (SCL-90-R) for parents (n = 88) and older children (age 19-23 yrs, n = 12), while children aged 4-18 (n = 94) were assessed using the Strengths and Difficulties Questionnaire (SDQ).

**Results:**

Thirty percent of the families had one parent with a high psychological distress score ("probable caseness" for a mental disorder), while only 4% of the children aged 10 - 23 years were considered as probable cases. In spite of this, there was an association between probable caseness in children and in fathers at T3. A significant negative paternal predictor for the children's mental health at T3 was the father's PTSD at arrival in Norway, while a positive predictor was the father's participation in a Norwegian network three years after arrival.

**Conclusions:**

Children of refugees cannot be globally considered at risk for mental health problems. However, the preceding PTSD in their fathers may constitute a specific risk for them.

## Introduction

Intergenerational transmission of trauma has been hypothesized to be an important determinant of the mental health of refugee children. Mental health consequences of parental trauma have been studied in the offspring of Holocaust survivors [1,2], and combat veterans [3,4], and there are some reports on the intergenerational transmission of trauma in refugee families, focusing on war-related traumatisation [5,6] or torture [7,8]. Additionally, like any other children, refugee children's mental health may be affected by affectively ill parents [9-11].

Reviews of studies of the mental health of offspring of Holocaust survivors have concluded that the non-clinical cohort of offspring does not seem to have more psychopathology than others [12,13]. Yehuda et al found, however, increased vulnerability for post-traumatic stress disorder (PTSD) and other psychiatric disorders in community studies of offspring of survivors, demonstrating that having a parent with PTSD may be one of the factors predisposing children to this vulnerability [14], especially if the parent was the mother [15].

Conflicting results are found also in studies of trans-generational effects of trauma on children of combat veterans. While some describe negative consequences of the fathers' PTSD on marital and family adjustment and parenting skills, resulting in increased emotional and behavioural problems in the children [3,4], others emphasise the bidirectional nature of the interaction between the traumatized individual and the family [16], or even report PTSD symptoms not to be significant predictors for family functioning across time [17].

There are several studies of refugee families, a large number investigating the intergenerational conflicts related to the different paces of acculturation between refugee parents and their children in the new culture [18-20] and the challenges faced by the first- and second-generation children in the resettlement countries [21].

Some studies underline the importance of the social network for the mental health and well-being for refugees [22]. Community studies of Vietnamese refugees in the US show the importance of social support from same ethnic communities. Contrary to findings from clinical studies [23], there was no association between support from the host-community and mental health. These studies suggest that the interplay between acculturation and mental health is multidimensional and results from the interaction of a network of factors [24]. Rousseau et al [25] describe the dual role of the extended family which can constitute an essential source of support, but also sometimes, especially for the second generation, may become a burden. In the Vietnamese community this appeared to be linked to demands for conformity and to the obligations toward the extended family.

Other studies focus on trauma, reporting mainly negative effects on the children, while a few studies [5,26] also depict that the transmission of family trauma may have dual effects, sometimes increasing vulnerability, but uncovering resilience and triggering possible positive transformation processes, included in the concept of posttraumatic growth [27]. However, these studies were mainly cross-sectional. We have not identified prospective studies where an adult refugee cohort has been followed for several years, including spouses and children born in the resettlement country.

The current study reports data from a longitudinal, prospective cohort study of Vietnamese refugees arriving in Norway in 1982 (T1), followed-up on in 1985 (T2) [28,29] and in 2005/06 (T3) [30]. At T3, we additionally included spouses and children born in Norway. The study focuses on the mental health of parents and their children who were born in Norway.

It provides an opportunity to study the long-term effects of parents' trauma, flight and mental health in the early resettlement phase on their offspring, born in the resettlement country.

**Figure 1:**
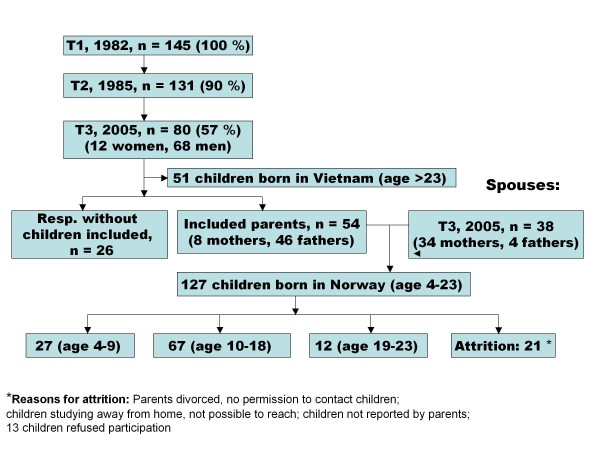
**Flow diagram of Vietnamese refugees, spouses and children included at T3**.

As most original respondents included at T3 were men (Figure 1), paternal predictors at T1 or T2 of the children's mental health at T3 were studied. Do the fathers' background, pre-migration trauma, and adverse events related to flight and exile have an impact on the mental health of their children? While only fathers could be included in the analyses of long-term consequences of parental trauma, all parents interviewed at T3 were included in the analyses of associations between the mental health at T3 in refugee parents and in their children born in exile.

Aims of the study

1. To study the association between the mental health of Vietnamese refugee parents and their children after 23 years resettlement.

2. To analyse paternal predictors for their children's mental health.

## Methods

The adult refugees included in the current report belonged to the surviving cohort of refugees that was originally included in a study on their arrival in Norway in 1982 (T1). The refugees had been rescued by chance from the South China Sea by Norwegian merchant vessels, and were given an offer to resettle in Norway. So, this original cohort may be regarded as a relatively unselected sample from the third wave of Vietnamese "boat people" who fled the Vietnamese communist regime after the war in Vietnam [29]. Figure 1 is a flow diagram of Vietnamese refugees, their spouses and their children included at T3 (2005/06).

### Design and procedures

A structured interview was administered in the respondents' home by the first and fifth authors at T3. Both mothers and fathers and their offspring aged 4-23 years were included.

**Figure 2:**
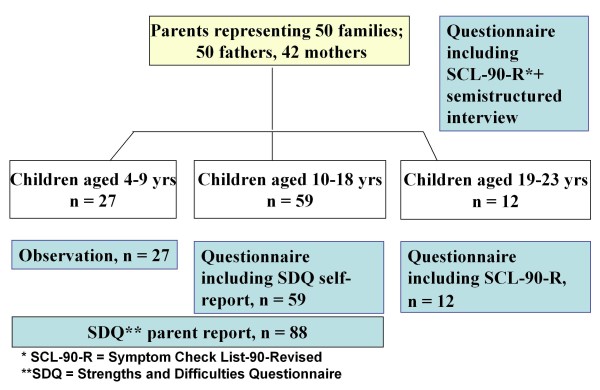
**Assessment of parents and children at T3**.

Parents were interviewed in Vietnamese; children aged 10 years or older, all fluent in Norwegian, were interviewed in Norwegian. The parents assessed their children aged 4-18 years (Figure 2).

The assessment of parents and children included structured self-report questionnaires and semi-structured interviews. The children sat apart from their parents while they filled in the questionnaire and during the interview.

Written information about the study was provided in Vietnamese and Norwegian. The parents consented for their children to be included in the study, and both the parents and their children aged 10-23 years signed a consent prior to the interviews. The study was approved by the Regional Committee for Medical Research Ethics and the Norwegian Social Science Data Services.

### Study populations

One or both *parents *of the children included in the current report were original respondents included in this study for the third time. The parents consisted of 42 mothers (8 original respondents) whose mean age was 40.3 years (SD 6.1) and 50 fathers (46 original respondents) whose mean age was 45.8 years (SD 5.4). Eight original respondents were married, representing four couples. All parents were Vietnamese, born in Vietnam.

Of the 127 *children *or offspring of the refugees, aged between 4 and 23 years, born in Norway and eligible for inclusion in the study, we were able to include 83.5%; 49 girls and 57 boys (mean age: 12.8 years, SD: 4.6 years). Figure 1 shows the reasons for attrition.

*Information from the parents *on the children's mental health was available for 88 of the children included in the study (age 4-18 years), mainly from the mother.

#### Population characteristics

The included parents represented 50 families; 41 couples participated, while an additional four couples were represented by one parent. There were five single-parent families (10%). None of the older children (age 19-23 years) had moved from their families.

The families lived in a geographically widespread area in the southern part of Norway, representing both urban and rural districts.

Table 1 shows socio-demographic variables for included parents at T3. Their main religious affiliation was Catholic (54%) or Buddhist (38%). The parents spoke mainly Vietnamese with each other (about 80%). With their children, 40% of the fathers and 31% of the mothers spoke mainly Vietnamese. The others used a combination of Vietnamese and Norwegian. A minor group spoke only Norwegian with their children (one mother and four fathers).

**Table 1:** Socio-demographic variables for parents and mental health for parents (Global Severity Index, GSI) and children (Strengths and Difficulties Questionnaire, SDQ, and GSI) at T3.

		Mother (n = 42)	Father (n = 50)
**Socio-demogr**.	Mean number of children	2.6 (SD1.0, range 1-5)	2.7 (SD 0.91, range 1-5)
	Total number of years education	11.7 (SD 4.0)	12.8 (SD 4.5)
	Employment	85% (n = 35)	77% (n = 47)
	Norwegian network		
	≤10 friends	64% (n = 18)	69% (n = 31)
	More than 10	36% (n = 10)	31% (n = 14)
	Vietnamese network		
	≤10 friends	29% (n = 8)	44% (n = 20)
	More than 10	71% (n = 20)	56% (n = 25)
**Mental health parents**			
Mean GSI		0.45 (SD 0.47)	0.50 (SD 0.51)

**Mental health children**			
Age 19-23 years	Mean GSI (n = 12)		0.37 (SD 0.38)
Age 10-18 years	SDQ, self-reports, mean total problem score (n = 59)		9.3 (SD 4.6)
	SDQ, parents reports, mean total problem score (n = 61)		9.1 (SD 6.0)
Age 4-9 years	SDQ parents reports, mean total problem score (n = 27)		8.9 (SD 5.0)

### Assessments

#### Socio-demographic variables

##### Parents

*Variables *in the self-report questionnaire included marital status, family re-union, presence of family in Norway, social network including Vietnamese and Norwegian friends, religious affiliation, total years of education, employment, and economic support. The variable "number of friends" (none, 1-2, 3-5, 6-10 or more than 10) was dichotomized to 10 or less vs. more than 10 friends, as studies show that the social perception of the social network (few or many) is more interesting than the precise number and exact frequency of contact [25].

Several of the socio-demographic variables were taken from two large population-based studies in Oslo conducted by the Norwegian Institute of Public Health http://www.fhi.no/tema/helseundersokelse/oslo/index.html, the University of Oslo (UiO) and the municipality of Oslo.

#### Mental health

##### Children aged between 4 and 18

The mental health of 94 children aged 4-18 was assessed using the SDQ http://www.sdqinfo.org[31,32], a brief behavioural screening questionnaire. The SDQ is translated into to a whole range of languages and is found to have reliable psychometric properties cross-culturally [33]. The self-report questionnaire was used for 59 of 67 children aged between 10 and 18, in accordance with a Norwegian study [34], with parent reports for 88 of the 94 children aged from 4 to 18. The SDQ consists of five subscales, each with five items, covering four problem areas (emotional, conduct, hyperactivity-inattention, and peer problems) and a fifth subscale assessing positive aspects of pro-social behaviour. A total difficulties score (0-40) was calculated by adding the four problem subscales scores, with each item being scored from 0 to 2 (not true, somewhat true, certainly true).

Cut-off points for the SDQ total score to define a 10% high risk group and an 80% low risk group are presented by Goodman http://www.sdqinfo.org. Since there are no culturally defined cut-off points, we chose to include both the 80^th ^and the 90^th ^percentile in the analyses of caseness, using the adjusted values from the Akershus study [34], a Norwegian population based study from 2001 including 36,465 school-children aged 9 - 19 years. In this way, we categorized the participants into a low-risk or normal group (below the 80^th ^percentile, total problem score 0-15), a borderline group (80^th^-90^th ^percentile, score 16-18), or a high-risk or abnormal group (above the 90^th ^percentile, score 19 and above). The cut-off scores were slightly lower for preadolescents (grades 5-8). The borderline group represented children with non-optimal functioning. For the children above the 90^th ^percentile we use the label "probable cases".

The findings from the study comparing the mental health of children aged 4 to18 with their Norwegian peers are reported in detail elsewhere [35].

##### Older children and parents

The mental health of 12 children aged 19 to 23 and all parents was scored using the Symptom Check List-90-Revised (SCL-90-R) [36], a widely used self-report rating scale for the measurement of psychological distress. The instrument is considered valid and reliable, and has been used in several studies of refugee mental health, both in its original form [37,38] and as the shorter Hopkins Check List-25 (HSCL-25) [39].

Ninety statements describing physical and psychiatric symptoms are evaluated using a five-point Likert scale ranging from "not at all" (0) to "extremely" (4). The Global Severity Index (GSI) is the mean score for all 90 items. The most commonly used cut-off point on the SCL-90-R to identify a psychiatric "case" is a GSI of 1.00 or more [40]. As there is no culturally defined cut-off for the GSI, we use the label "probable caseness", parallel to the definition of caseness in children.

The SCL-90-R was translated into Vietnamese and Norwegian, and the same translation as used in the previous two studies (T1 and T2) was used.

The findings from the study of the mental health of Vietnamese refugees in Norway after 23 years in exile are published elsewhere [30].

#### Family cohesion

The self-report questionnaire for children covered a wide range of themes, including family and friends. Questions were taken from two large Norwegian population-based youth studies (NOVA and the Oslo Health Study, Ung-HUBRO), and from the study "Adolescent mental health in multicultural context" [41].

For the present study only items concerning family climate were analysed, to control for confounders. The variable called "family cohesion" was computed by combining six variables on the respondents' evaluation of the importance of satisfying the family's needs before their own, avoiding quarrelling, giving preference to the family's needs, sharing belongings, sharing money with the family and the importance of fulfilling the family's expectations, each to be graded from 1 (little or no importance) to 4 (high importance). The children's cohesion index had a good internal consistency (Cronbach's alpha 0.84). The reversed value of the single item "importance of avoiding quarrelling", rated on a Likert scale from 1 (very important) to 4 (not important at all), was used as an indirect indication of aggression. These confounding variables were chosen because they are reported to influence the children's mental health in families with traumatized parents [3,42]. The variables were in the questionnaire, and chosen as a measure of family cohesion during the analyses and discussion of findings.

### Trauma exposure and PTSD in the fathers

Trauma prior to and during the escape was included, and an additive index combining being wounded in the war, having been incarcerated in prison or a concentration camp for one year or more, and having been in great danger before the escape represented "extreme traumatic stress before the escape" (minimum score 0, maximum 3).

Post-traumatic stress at T1/T2 was reported for those fulfilling the diagnostic criteria for a post-traumatic stress disorder (PTSD) according to the DSM-III criteria, but also for respondents with core criteria symptoms, without satisfying the whole set of criteria, as a combined variable of full or partial PTSD [43,44]. This combined variable was used in all the analyses.

At T2 life events after resettlement and their impact were recorded and the dichotomized variable (no high-impact events or one or more high-impact events at T2) was included in the analyses.

### Statistical analysis

Except for some descriptive information regarding parents, all analyses were based on data of the children, with their parents' characteristics included as variables at the child level. A number of categorical variables were dichotomized to obtain the same categorization as at T1/T2. Marginal tests of homogeneity and McNemar's test, and chi-squared, Mann-Whitney U, and t tests were used for paired and two-sample comparisons. Intra-class correlations were used to measure agreement between continuous variables in children and their parents.

The children's mental health at T3 was investigated using simple and multiple linear regression analyses, with the self-reported total problem scores (SDQ, n = 59) at T3 as the dependent variable, by pre-specified independent parent variables. Paternal variables from T1, and if not included at T1, then at T2, were used. As there was a majority of men among the refugees (only eight mothers were original respondents, Figure 1), the regression analyses were based only on characteristics of the original respondents who were fathers. Regression analyses used methods taking clustering of siblings within families into account, using the generalised estimating equations (GEE) procedure [45]. Covariates included information on psychological distress, self-reported health, trauma prior to and during the escape, education and employment, and social network, including family, Vietnamese, and Norwegian friends.

The variables included in the multivariate analyses were chosen based on what has been discussed as important factors for mental health outcome in children.

For univariate analyses of the association of the mental health of parents and children at T3 we included all 106 children, while for the multivariate analysis we included the 59 children with self-reported mental health (SDQ). In some families, there were family-members who did not want to participate, or participated in parts of the assessments. Hence the numbers of respondents in the different analyses varies.

The level of significance was set at .05. Statistical tendencies were reported when p < .10. All analyses used SPSS versions 15 and 17 (SPSS Inc, Chicago, IL, USA) and R (The R Foundation for Statistical Computing, Vienna, Austria) for GEE analyses.

## Results

### 1. Mental health of parents and children at T3

Table 1 shows the mental health of parents and children at T3. One-fifth of the fathers were identified as probable cases, with a GSI ≥1.00 (n = 10, 20.4%), while only one-tenth of the mothers were probable cases (n = 4, 9.8%) at T3. No family had two parents scoring as probable cases. Consequently, 28.0% of the families (n = 14) had one parent scoring as a probable case, and 27.4% (n = 29) of all children were living with one parent scoring high on psychological distress.

In the age group 10-18 two children (3.4%) scored as probable cases according to the 90^th ^percentile distribution on the self-report SDQ. Using the 80^th ^percentile as a cut-off value, we found nine children (15.3%) with borderline or abnormal values. Among the offspring aged 19-23, one of 12 (8.3%) had a GSI score indicating a probable case. Thus, the total group of children scoring as probable cases was 4.2% (n = 3) and 14.1% (n = 10) when the group with borderline values was included.

In the youngest age group (4-9 years) one child (3.7%) was categorized by parent report as a probable case, above the 90^th ^percentile, according to the British cut-off values.

### 2. Associations between children's and their parents' mental health at T3

Except for the significant association between the older children's GSI and their fathers GS1, there were no correlations between the parents' GSI and the children's parent- or self-rated total problems (SDQ and GSI), as shown in Table 2.

**Table 2:** Intraclass correlations for mental health of parents (Global Severity Index, GSI) and children (Strengths and Difficulties Questionnaire, SDQ) at T1 (fathers) and T3 (parents and children).

	Mother's GSI T3, n = 38	Father's GSI T3, n = 48	Father's GSI T1, n = 45	SDQ self-reports **10-18 yrs**^**b**^
**Father's GSI T3**	-.006			
**Older children's GSI T3, n = 12**	.607	.623^**a**^	-.134	
**SDQ self-reports (10-18), n = 59**	.043	-.015	.015	
**SDQ parent reports (4-18 yrs), n = 88**	.083	.010	.011	.153

^**a **^p = .020

^**b **^54 cases analysed

There was a significant association between probable cases in the combined group of children (two oldest age groups) and probable caseness in fathers (McNemar's test, p = .013), while there was no association with mothers' probable caseness. Including the group with borderline SDQ values, we found no significant association with parents' probable caseness.

Other parental variables at T3, such as education, employment, and social network, were not associated with children's mental health at T3, except for fathers who had more than 10 family members in Norway, with a lower self-reported total problem mean score in the children aged 10 - 18 years (7.2 vs.10.1, n = 15 vs. 45, Mann-Whitney U test, p = .026).

### 3. Prediction analyses

#### A. Univariate analyses

In univariate analyses we found no significant correlation between the fathers' GSI at arrival (T1) and their children's self-reported mental health at T3 (SDQ or GSI) (Table 2), nor any significant association between fathers' GSI at T1 and probable caseness in their children, the oldest age groups included.

Analysing the fathers scoring above cut-off for probable caseness according to the GSI at T1, we found no association with their children's self-reported mental health (SDQ or GSI), but a significant association with probable caseness in their children aged 10 and above (McNemar, p = .013).

Analysing the association between other relevant predictors from the fathers at T1 or T2 (described in methods) and self-reported total problem scores in children at T3, corrected for siblings in the families (Table 3), we found the fathers' PTSD at arrival to be a significant negative predictor, while participation in a Norwegian network after three years was a significant positive predictor for the children's mental health. We found no association between the children's total problems and their fathers' trauma variables, neither single variables nor the additive index for extreme trauma.

**Table 3:** Univariate regression analyses using gee, correcting for siblings.

	Self-report problems	
Variables father T1/T2	**Estimate**^**a **^**(95% CI)**	p-values
GS1^b ^T1	1.85 (-2.2, 5.92)	.37
Years education before arrival	.097 (-0.27, 0.46)	.60
Additive stress T1	.075 (-2.43, 2.58)	.95
PTSD T1		
- not present (n = 50)	1	
- total or partial (n = 4)	7.23 (2.43, 12.04)	.003
Hi-impact events T2		
- no events (n = 43)	1	
- events (n = 6)	-2.71 (-6.40, 0.98)	.15
Close confidant T1		
- yes (n = 25)	1	
- no (n = 28)	2.29 (0.15, 4.73)	.066
Employment T2		
- no (n = 38)	1	
- yes (n = 16)	0.76 (-2.17, 3.68)	.61
Vietnamese network T2		
≤10 friends (n = 25)	1	
>10 friends (n = 24)	0.32 (-2.26, 2.90)	.81
Norwegian network T2		
≤10 friends (n = 47)	1	
>10 friends (n = 2)	-6.19 (-8.63, -3.76)	<.001

Relationships between mean self-reported total problem scores in 59 children aged 10 - 18 at T3 and paternal variables at T1/T2.

^a ^For continuous variables regression coefficients, for categorical variables differences

^b ^GSI = Global Severity Index of the Symptom Check List-90-R

We checked possible relationships between dichotomous paternal PTSD at arrival versus variables on family environment, such as family cohesion, based on reports from both the child and the father by two-sample t tests, finding no significant associations.

At T1, 17.4% (n = 8) of the fathers had full or partial PTSD, while 28.3% (n = 13) had full or partial PTSD at T1 and/or at T2. At T3 the rate of PTSD was still high (15.2%, n = 7). Only one child included in the analyses had a father with PTSD both at arrival and at the second follow-up. At T1 there was no significant association between PTSD and probable caseness in the fathers.

#### B. Multivariate analysis

We then performed a multiple regression analysis with the children's self-reported mental health as a dependent variable and variables from the fathers at T1/T2 as covariates, correcting for siblings (Table 4). A significant negative predictor for the mental health of the children at T3 was the fathers' PTSD at arrival in Norway.

**Table 4:** Multiple regression analysis of paternal predictors of children's self-reported mental health (SDQ), n = 59, aged 10 - 18, using gee, correcting for siblings

Variable	Regression coefficients	p	Confidence interval
Child's age at inclusion	0.078	0.85	-0.70, 0.86
Child's gender^a^	-0.83	0.62	-4.14, 2.48
GSI ^b ^T1	-0.77	0.74	-5.38, 3.83
PTSD^c ^T1	7.84	0.003	2.68, 13.00
Norwegian friends^d ^T2	-4.79	0.004	-8.02, -1.56

^a ^Gender: (Boy = 1, girl = 2), reference category "boy"

^b ^GSI = Global Severity Index of the Symptom Check List-90-R

^c ^PTSD T1: (No PTSD = 0, full or partial PTSD = 1), reference category "no PTSD"

^d ^Norwegian friends at T2: (10 or less = 0, more than 10 = 1), reference category "10 or less"

## Discussion

The main findings from the study were twofold. First, at T3 30% of the Vietnamese families had one parent with a high psychological distress score, categorized as a probable case according to the GSI, while only 4% of the children were considered as probable cases according to the SDQ (ages 10-18) or GSI (ages 19-23). In spite of the generally low level of child psychopathology, there was an association between probable caseness in offspring and in fathers at T3.

Second, traumatic experiences without PTSD at T1 did not predict mental problems among the offspring. A significant paternal predictor was PTSD at arrival, not the general level of psychological distress.

It is important to underline that Norwegian Vietnamese children, as a group, report less psychological distress than their Norwegian peers [35], in spite of the high exposure to premigratory adversity and in spite of the fact that 30% of the fathers reported partial or total PTSD at T1/T2. Taken together, these two sets of analyses suggest that there is simultaneously an overall resilience in the second generation, while mental health problems of the parents may be associated with subsequent psychopathology in a subset of more vulnerable children.

This complex picture is in line with empirical studies of the mental health of children, and the grandchildren of Holocaust survivors [46]. While clinical studies have repeatedly confirmed the hypothesis of traumatic transmission, well designed general population studies have emphasized resilience [47,48]. In his interpretation of this surprising resilience, Sigal [46] proposes a complex model that takes into account endowment, temperament, family, and environmental factors before and after persecution [49].

The association between fathers' PTSD at arrival in Norway and their children's mental health 23 years later, suggests a specific vulnerability of a subgroup of children that raises a few hypotheses [50]. First, this association may simply reflect the fact that parental mental health is an important predictor for the mental health of children in general [51,52], and of refugee children in particular [53]. They must simultaneously handle the tasks of developing into adult beings and adjusting to two cultures, the culture of their parents and the culture of the settlement country that they encounter in school and with friends [54]. For a subgroup of children, these tasks may exceed their coping resources.

Second, living with a traumatized parent can be a very severe and threatening circumstance [26], disrupting family life and threatening the fundamental secure base needed for the child's adequate psychological development of secure attachment. Several studies of Vietnam veterans document the disruption of the family environment [3,4], in parallel with a recent study of Cambodian refugee families investigating the relation between PTSD and long-term family dysfunction after Pol Pot [55]. In the reported study however, the absence of association between a father's PTSD and family environment variables does not support this hypothesis. The fathers' capacity for attachment, represented by the presence of a close confidant at arrival [29], may have compensated for some of the problems in the aftermath of trauma. This is in line with a study of the role of attachment for adjustment to trauma [56]. Another factor explaining the positive findings in the study may be the spouses' possible buffering effect in the families, as described in the literature [42,57,58].

Third, our results underline a possible gender effect in the transmission, highlighted by the significant association between probable caseness in fathers and children at T3. The association between the fathers', rather than the mother's, probable caseness at T3 contrasts with findings from a meta-analysis by Connell [58], who found the association between maternal psychopathology and the presence of problems in the children to be stronger than between paternal psychopathology and children's problems. The cultural background of the families in our study may, however, account for this difference. In a study comparing Cambodian refugee fathers' and mothers' reports of symptoms for their children, Rousseau et al. [59] describe a stronger father-child than mother-child agreement around symptoms in Cambodian children and adolescents, especially with regard to internalizing symptoms. While the role of women in restoring or maintaining family harmony when faced with emotional difficulties may be responsible for some underreporting on the part of mothers [60], these results also support the hypothesis of a strong emotional bond between fathers and children in the South-East Asian refugees.

Fourth, genetic vulnerability, in combination with early environmental factors, such as the quality of parent-offspring interactions, can influence development and partly explain variations in mental health, including vulnerability or resilience [61-63].

The included children were all born in exile, as a parallel to Cambodian refugee youth from traumatized families, studied by Rousseau et al in Canada [5]. Parents' trauma prior to the birth of a child seemed to play a protective role when the child reached adolescence. The youths' low levels of behavioural problems were interpreted as both a reflection of the internalization of cultural standards of conduct and as overcompensation, caused by the children's inherited obligation to succeed for the sake of those who had died. In our study, several youth in the interviews described their indebtedness to their parents for their efforts to create a good life in Norway.

This indebtedness to their parents and their responsibility to become successful on behalf of the family [64] may, however, in the long run represent a burden to the second generation. Consequently, the long-term adaptation across generations should be studied further.

Two significant findings underline the importance of the social network for the children's mental health; namely the lower problem score in children of fathers with a large family network in Norway at T3, and the fathers' early contact with the Norwegian population as a positive predictor for the children's mental health 20 years later. Although there is no clear answer to the question of the relationship between acculturation status and mental health [23,24,65,66], our finding of a simultaneous integration into a Vietnamese and a Norwegian network seems to indicate a mental health advantage for the children included, pointing to elements of resilience in the families, although the small sample size warrants a cautious interpretation of the findings.

A clinical implication related to the finding of PTSD as a predictor for the children's mental health is the importance of an awareness of the parents' trauma-related backgrounds, both within the community health services and in terms of specialist mental health services. Therefore, a family history should include questions about pre-flight traumatisation, traumatisation during the flight, and traumatization or adverse events experienced as asylum-seekers. Family counselling of traumatized families should be included in the health services made available to refugees. On the other hand, the finding of resilience shows that the refugees have a range of coping mechanism. Consequently, a focus on social support and providing opportunities for acculturation for newcomers may be considered as an important approach.

### Strengths and limitations

Because this prospective follow-up study was of considerable length, its longitudinal design is a major strength, allowing analyses of paternal predictors from their first few years in Norway. Retrospective data on paternal trauma related to war and flight were reported soon after arrival, in contrast to some other studies where trauma was reported after several years, or even by the offspring [14]. The personal follow-up design of the study was strengthened by a culturally relevant approach achieved through the collaboration with the Vietnamese co-researcher. As he was responsible for making contact with the families, his efforts contributed to the relatively high inclusion rate of children.

There are, however, important limitations to be considered. The original study sample is small, preventing analyses of children with two original respondents as parents compared to those who had only one parent included from 1982. As most of the original respondents were men, it was not possible to compare gender issues, such as paternal vs. maternal predictors of the children's mental health. Another consequence of the small sample is that the number of fathers with PTSD at T1 is low. Cautious interpretations of the findings are therefore warranted.

Although the lack of longitudinal data on the mothers' mental health is an important limitation, the fact that the mental health of mothers at T3 is better in terms of both GSI and probable caseness, and the absence of an association between mothers' mental health at T3 and children's mental health (which is relatively surprising), confirm the presence of an important gender effect in the parents' mental health. We do, however, acknowledge the limitation represented by the few mothers scoring with high psychological distress ("cases").

Further, the lack of cultural validation of the assessment tools is a general problem that is not limited to this study, and represents a major challenge in transcultural research. An aspect of that problem is the caseness determination in the SDQ, without a culturally defined cut-off point. Predicting a risk group for children (the 80^th^-90^th ^percentile of the SDQ) would indirectly signify "normalization" of the risk.

The study did not include clinical diagnostic interviews of parents and children at T3, which limited the possibility of comparisons with some other studies.

Because of the wide age range, it was necessary to assess the children using two different methods. The choice of the SDQ as an assessment tool was based on the need for a short questionnaire, to limit the burden on the parents in the interviews. Consequently, the lack of uniform methodology for all respondents, although necessary, must be considered a limitation. We chose to use the self-reported SDQ in the regression analyses. As a group, the children of Vietnamese refugees are more highly acculturated than their parents [67]. Consequently, using self-reports as the dependent variable may be considered as more culturally relevant than using the parents' reports.

Even so, the refugees studied at T3 were considered to be a representative sample of the third wave of boat refugees who arrived in Norway in 1982 [68]. The major characteristics of the parents included in the study were the same as those in the group who did not have children born in Norway. Consequently, the children may be considered a representative sample of second-generation Vietnamese in Norway, who belonged to this group of refugees. Although incomplete, this longitudinal set of data provides a very important insight into the possible long-term consequences of PTSD in refugee parents on their children.

## Conclusion

The simultaneous finding of a low level of symptoms in the children as a group, and of a specific association between fathers' PTSD on arrival in Norway and their children's mental health, suggests that the children of refugees cannot be globally considered as at risk for mental health problems. However, the preceding PTSD in their fathers may constitute a specific risk for them. Fathers' early participation in a Norwegian network and a large family network in Norway seems to represent mental health advantages for their children.

## Competing interests

The authors declare that they have no competing interests.

## Authors' contributions

ABV participated in planning of the study, carried out the interviews, conducted the statistical analyses, discussed the results and prepared the manuscript. PHT participated in planning of the study, discussed the results and the draft. CR discussed the results and the draft. TWL conducted the statistical analyses and discussed the results. TVT participated in planning the study, carried out the interviews and discussed the results. EH performed the two first studies of the Vietnamese refugees (1982 and 1985), participated in planning of the current study, and discussed the results and the draft.

All authors read and approved the final manuscript.
